# Molecular Research in Rice

**DOI:** 10.3390/ijms241210063

**Published:** 2023-06-13

**Authors:** Prasanta K. Subudhi

**Affiliations:** School of Plant, Environmental, and Soil Sciences, Louisiana State University, Baton Rouge, LA 70803, USA; psubudhi@agcenter.lsu.edu; Tel.: +1-2255781303

## 1. Introduction

Rice is the most important source of nutrition for approximately half of the human population [1]. While it fulfills 21% of the energy needs of the global population, it is the lifeline for people in Southeast Asian countries, contributing to 76% of their caloric intake [2]. Social stability, food security, and economic development in those countries depend on increased rice productivity due to the major contribution of rice toward their economies. Rice consumption will continue to increase due to several factors, such as population growth, dietary changes, increased economic conditions, and yield enhancement [3]. Since the global population is expected to reach 9 billion by 2050, increased rice production will be vital to prevent food crises in the future.

In addition to global population growth, climate change and the plateauing of the rice yield have increased the urgency to boost rice production. Rice farming in many countries is threatened by a number of biotic and abiotic stresses due to climate change. Innovative strategies should be devised to design novel, high-yielding, and climate-resilient genotypes to improve the sustainability of rice farming. It is imperative to explore the genes and the regulatory network of agronomically important traits such as yield and yield component traits, tolerance to various biotic and abiotic stresses, and grain quality traits. Appropriate molecular tools should be developed for use in breeding programs to accumulate desirable traits and genes. Due to spectacular advances in molecular biology, genetic engineering, and various omics fields, these goals can be realized through the application of new molecular tools and technologies.

There are many morphological and physiological traits which require improvement to enhance the genetic yield potential of every crop, including rice. In the case of ideotype breeding, for example, researchers visualize rice plant architecture, and then traits with direct or indirect influence on crop productivity are continuously improved [4]. The current Special Issue, entitled “Molecular Research in Rice”, of the *International Journal of Molecular Sciences* encompasses a collection of nine original research articles and one review unraveling the molecular basis of some key agronomically important attributes, such as salinity tolerance, flowering, tiller and leaf angle, grain weight, and tolerance to brown plant hopper and white backed plant hopper, using advanced molecular tools.

### 1.1. Studies Deciphering Molecular Basis of Grain Quality Traits

Understanding the molecular basis of grain size variation is necessary for breeding high-yielding rice varieties. Since several QTL/genes for grain size have been identified, there are opportunities to apply marker-assisted selection for rice yield improvement. Jiang et al. [5] provided an update on the QTLs for grain length and grain width, as well as grain thickness. This review also discussed various regulatory pathways controlling grain size, such as G protein signaling, the mitogen-activated protein kinase (MAPK) signaling pathway, the ubiquitin–proteasome pathway, phytohormone signaling, transcriptional regulatory factors, etc., and their interactions. It was also suggested that the application of genome editing and marker-assisted pyramiding of desirable alleles should constitute important strategies for enhancing the yield potential of rice varieties.

The discovery of novel genetic variation is necessary for success in plant breeding programs. Grain weight is an important component trait for increased yield. Zuo et al. [6] validated a candidate gene, OsMADS56, encoding a MADS-box transcription factor. This was identified as the most important candidate gene in the grain weight QTL qTGW10-20.8 region in an earlier study. In addition to validation using the near-isogenic lines, both knockout and overexpression strategies were employed. While the OsMADS56 knockout mutant plants resulted with reduced grain sizes due to reduced expression, the overexpressing plants, under a strong constitutive promoter, had higher grain weight. Another important finding from this study was that the Teqing type allele of OsMADS56, which was predominantly present in cultivated rice, should be used for molecular breeding to improve rice productivity.

The investigation of Liu et al. [7] revealed that OsMADS1 regulates grain quality by regulating starch and storage protein metabolism in rice. However, the regulatory mechanism has yet to be elucidated. This study was carried out because findings on the role of this gene-regulating grain quality have been contradictory in earlier studies. Using a mutant of OsMADS1 with chalky endosperm, abnormal grain morphology, low starch content, and loose packing of starch granules, but with high protein content, this study demonstrated the OsMADS1-coordinated gene expressions and regulatory network of starch and storage protein metabolisms. Linkage mapping using F_2_ and F_3_ segregating populations developed from a cross of a mutant and Nipponbare revealed that the starch and storage protein contents were controlled by different genes. Further analysis of the segregating populations indicated linkage of grain quality with its grain shape and, possibly, by the pleiotropic effects of OsMADS1. Complementing the OsMADS1 mutant with its wild-type allele recovered the normal grain shape, similar to Nipponbare. Seed-specific RNAi of the OsMADS1 gene confirmed the role of this gene in regulating grain quality. Mapping by sequencing the two bulks with contrasting grain quality suggested the role of this gene in regulating seed development and grain quality. Like other MADS-box transcription factors, it was localized in the nucleus. Transcriptomic analysis further suggested that the difference in grain quality between the mutant and its normal counterpart was due to differences in the expression patterns of genes associated with starch and storage protein biosynthesis. These findings open up possibilities to improve grain quality in rice by manipulating the expression of OsMADS1.

### 1.2. Characterization of Genes/QTLs Controlling Tiller Angle, Leaf Angle, and Crown Root Development

The design of a favorable plant architecture is essential for achieving high productivity in rice crops. Among many traits, an appropriate tiller angle and appropriate root characteristics are necessary for crop density and growth and development, respectively. In a doubled haploid population developed from an *indica* × *japonica* cross, Zhao et al. [8] identified a major stable QTL region on chromosome 9, which overlapped with 2 QTLs each for tiller angle and tiller crown width. Based on an expression analysis of genes in the region, an auxin-responsive protein family gene, OsSAURq9, was selected as the main candidate for further investigation of the molecular basis of the tiller angle.

Hu et al. [9] isolated and characterized a temperature-sensitive mutant which inhibits crown root development in rice at high temperatures. Crown roots help in absorbing water and nutrients, and any abnormality may impair plant growth and development. Using a map-based cloning strategy, the SCR8 gene was mapped to a 126 kb region on chromosome 8, but only LOC_Os08g14850, encoding a CC-NBS-LRR protein, showed sequence variation. There was lower expression under a low temperature and enhanced resistance to *Xanthomonas oryzae*. This study demonstrated that a mutation in this novel gene was responsible for the defective crown root while activating the defense response system.

Jang et al. [10] investigated the molecular regulation of two important traits: leaf angle and grain size. The leaf angle determines the photosynthetic efficiency of rice plants, and erect-leaved varieties enhance the rice grain yield by increasing the planting density. This study discussed the functional characterization of two members of a bHLH transcription factor family called OsBC1, OsBCL1 and OsBCL2, by overexpressing them under the control of a OsBUL1 promoter in both *Arabidopsis* and rice. These two genes were responsible for increased leaf angles and grain sizes due to cell expansion, and partially due to increased gibberellin (GA3) synthesis. It was concluded that both OsBCL1 and OsBCL2 are positive regulators of cell elongation/expansion. The findings from this study can be exploited to develop rice plants with reduced leaf angles by decreasing the expression of OsBC genes using lamina joint-specific promoters.

### 1.3. A Next-Generation Sequencing-Based Study Provided Insights into the Flowering Mechanism

An enhanced understanding of flowering time is necessary to develop varieties with local adaptability and improved yield in rice. The molecular basis of this important trait helps to design uniform maturing varieties with photoinsensitivity in order to expand the cultivation of rice to large geographic areas. In a study by Garcia et al. [11], genes responsible for flowering and their interactions were investigated through a comparison of the whole genome and transcriptome of weedy rice with cultivated rice. Due to the presence of latent genetic variation for flowering [12], photoinsensitive weedy rice accession harboring photoperiod-responsive genes, including the major locus *Hd1,* was used as a model to discover a new genetic mechanism for flowering in rice. The authors used various strategies. Firstly, the de novo assembly of unaligned sequences resulted in two weedy rice-specific genes associated with flowering. A comparison of variants of the weedy rice with the 3k rice genome dataset identified unique variants within the flowering time QTLs. Many differentially expressed genes (DEGs), identified from the comparison of the global transcriptomes of rice leaves collected from the short-day (SD) and long-day (LD) conditions, were congruent to some flowering QTLs. Some of these DEGs, such as *Hd1*, *OsMADS56*, *Hd3a,* and *RFT1,* had unique variants in weedy rice. Alternate splicing (AS) events in genes were influenced by genotype and day length variation. It was concluded that high-impact variants of important flowering genes may be responsible for flowering time variation in weedy rice. Based on the comparison of the whole-genome and RNA-sequence data of both cultivated and weedy rice under LD and SD conditions, the expression pattern and AS events led to the discovery of *OsMADS56* as the main determinant for the day-neutral flowering response in weedy rice. It was expressed in weedy rice, but not in cultivated rice, under both LD and SD conditions.

### 1.4. Genome-Wide Association Study Discovered Candidate Genes for Salinity Tolerance

Climate change-induced abiotic stresses are now major constraints for crop production. Among these stresses, salinity is making productive farmlands unsuitable for rice farming in many parts of the world. The genetic complexity of salt tolerance in rice is due to the involvement of many genes and their interactions, which control many morphological, physiological, and biochemical attributes. A genome-wide association study was performed on a panel of Thai rice genotypes to dissect the genetic basis of salt tolerance at the seedling stage [13]. Out of 25 predicted genes, 11 of them were congruent with the earlier reported QTLs for salt tolerance. One such predicted gene, *OsCRN* (LOC_Os05g22260), which was found to interact with the gene *AtSKIP,* conferring osmotic tolerance under salt stress, was characterized. The *Arabidopsis* mutant, with a T-DNA insertion in the orthologous gene, showed enhanced salt tolerance due to increased relative water content, cell membrane stability, and photosynthetic pigments. This observation was further supported by the enhanced susceptibility to salt stress by ectopic expression of the *OsCRN* genes in *Arabidopsis* transgenic plants. There was also lower expression of this gene in salt-tolerant rice lines compared with salt-susceptible lines under exposure to salt stress. This study led to the discovery of *OsCRN* as a negative regulator of salt tolerance, a finding which can be used to develop salt-tolerant varieties in rice breeding programs.

### 1.5. Role of Plant Hormones and Volatile Organic Compounds in Controlling Planthopper Infestation

Both white-backed planthoppers (WBPHs) and brown plant hoppers (BPH) cause serious damage to rice crop, particularly in countries in Asia. Hormones play a major role in regulating tolerance to a variety of biotic stresses. Some important hormones are salicylic acid (SA), jasmonate (JA), ethylene (ET), and gibberellic acid (GA). Asif et al. [14] investigated whether the application of exogenous hormones (gibberellic acid and methyl jasmonate) can resist WBPH infestation by reducing oxidative stress through regulation of the antioxidant defense mechanism and endogenous hormonal cross talk. After the application of this hormone on WBPH-infested rice plants, the authors measured the accumulation of antioxidants and hydrogen peroxide, recouping of affected plants, and cell death rate, along with the relative expression of genes involved in hormone biosynthesis. This study demonstrated that exogenous application of GA provided better protection to rice plants against WBPH compared with the MeJA.

The brown plant hopper is a devastating insect that can wipe out an entire rice crop. Although many BPH-resistant genes have been identified and deployed, breakdown of such resistance genes has been observed due to the evolution of virulent biotypes. Therefore, new sources of resistance to BPH are a continuous requirement of the rice breeding program. In this study [15], a major effect-resistance gene, *Bph45,* located on chromosome 4, was identified from *Oryza nivara* by genetic mapping. A near-isogenic line (NIL) for this gene in a *japonica* varietal background was compared with its recurrent parent with respect to the emitted volatile organic compounds (VOCs) in order to understand the underlying resistance mechanism. Among the VOCs, a high amount of limonene was responsible for attracting the BPH in the susceptible lines. The reduced amount of limonene emitted in NILs for resistant genes *Bph20* and *Bph18/Bph32* compared with the susceptible recurrent parent IR24 also supported the key role of limonene, in impacting the antixenosis effect against BPH infestation. This new *Bph45* gene will be useful for pyramiding with genes associated with different resistance mechanisms, to develop durable BPH-resistant rice varieties.

## 2. Conclusions and Future Perspectives

Compared with other food crops, rice is at the forefront of molecular genetics and genomics research. The availability of genome sequences, the abundant genetic variation among the world’s germplasms, the smaller genome size, and the diploid genome have been instrumental in making rice an ideal model for genomic and genetic research among all members of the grass family. Despite enormous advances in rice genetics and genomics, an enhanced understanding of many agronomically important traits, such as yield and yield component traits and tolerance to biotic and abiotic stresses, is still needed to break the yield plateau by developing the next generation of high-yielding varieties with climate resilience. The resequencing of diverse rice accessions boosted both basic research and applied breeding activities [16]. The collection of articles in this Special Issue provided some insights into the molecular basis of several important agronomic traits, which may be helpful in designing a novel rice ideotype with high yield potential and enhanced adaptability to climate change using modern genomics tools such as marker-assisted selection and genome editing. Due to its genetic closeness to major cereal crops, advances in molecular research in rice can be applied for the genetic improvement of other cereal crops.
